# Gutta Percha for Separating and Regulating Teeth

**Published:** 1860-07

**Authors:** B. Wood


### GUTTA PERCHA FOR SEPARATING AND REGULATING TEETH.

BY B. WOOD.

Among the many uses of gutta percha I have found it serviceable for making semi-elastic bands and ligatures applicable in separating and regulating the teeth, answering the purpose in many cases better than any thing else I have tried. Its usefulness for this depends upon a singular property.

If we cut a narrow strip of sheet gutta percha, say Slayton's, as prepared for temporary sets, and gradually stretch it with the fingers, it will be found to *draw out* from two and a half to three times its original length; at which point it resists any further *permanent* extension. It is now quite elastic, and, in consequence of the condensation and consolidation of its fibres by this tension, has acquired such strength as to defy the efforts of the fingers to pull it apart; whereas prior to this manipulation a light jerk would have snapped it in two, although of nearly thrice its present thickness—and for this reason it requires to be stretched gradually and evenly at the first.

In this condition it is ready for use, possessing strength sufficient, and the desirable amount of elasticity. By having sheets of different thicknesses we can thus obtain strips and bands to suit particular cases. For separating or wedging teeth a strip is drawn between them and cut off at either side. It is readily applied, and is neater, and more comfortable to the patient than wood, tape or rubber. It has sufficient firmness and elasticity to keep its place and exert constant pressure, while its elasticity is not so great as to produce undue irritation beyond our control. India rubber loosely drawn between the teeth fails to exert due pressure and easily slips out of place. If drawn tightly, the strain produces irritation, and *more work* is apt to be done by it than we intended; we can not regulate nor always foresee the result. On the other hand, tape, wood, &c., are too hard and unyielding.

This defect of rubber may, it is true, be obviated to some extent by using it in the form of rings of the desired thickness which shall be self-sustained by encircling the teeth, and which are also readily applied in cases that might otherwise prove troublesome, by the simple expedient of carrying the ring to its place by the means of two strings passed through it on either side, so as to stretch and adjust it as desired—an expedient which will prove useful to have mentioned if there are any who have not hit upon it. But rings are not always adapted to the case, and for front teeth they are objectionable on the score of appearance.

The gutta percha wedges are so pliant and so yielding as to be worn with little or no discomfort; they possess sufficient elasticity to do the work required of them without danger of overdoing it; and they present a body and surface that generally ensure their remaining *in situ*, or if there be danger of working out they may be secured at once by blunting or *riveting* the protruding ends with the headed point of a plugger.

In like manner these bands are well suited for regulating teeth, affording a frequently needed substitute for gum elastic bands on the one hand, and non-elastic ligatures on the other, being applied as above indicated, or in various ways, according to the nature of the case, which will readily suggest themselves to the operator—upon which I need not dwell in this, only designing to call attention to the qualities of gutta percha for the purposes referred to. The best material should be employed. And doubtless it might be prepared so as to answer these and similar ends better than any preparation of it now to be had.

